# Unveiling the Unlikely: Extraskeletal Ewing Sarcoma Masquerading as Gastrointestinal Bleeding

**DOI:** 10.7759/cureus.57109

**Published:** 2024-03-28

**Authors:** Bryan M Greenfield, Madeleine A Wilson, Kyle J Schulte, Stephen Silverstein

**Affiliations:** 1 Primary Care Medicine, Nova Southeastern University Dr. Kiran C. Patel College of Allopathic Medicine, Fort Lauderdale, USA; 2 Family Practice, MyCare Medical, Pompano Beach, USA

**Keywords:** iron deficiency anemia (ida), small bowel tumor, iron-refractory iron deficiency anemia, obscure gi bleed, extraskeletal ewing's sarcoma, extraskeletal ewing sarcoma

## Abstract

While Ewing sarcoma is traditionally a malignant tumor of bone, it may uncommonly present extra-skeletally, leading to an array of puzzling presentations depending on the tissue involved. Here, we describe the case of a 66-year-old man who presented to the primary care office for evaluation of intermittent melena. He ultimately underwent capsule endoscopy and developed a secondary small bowel obstruction, unveiling his neoplasm. The tumor was then resected and managed with surveillance only, and the patient remains without evidence of disease after four years of follow-up.

## Introduction

Ewing sarcoma is a highly malignant neuroectodermal small cell tumor most commonly affecting children and adolescents, with primary tumor locations in the femur, long axial bones, and pelvis [1]. Extraskeletal Ewing sarcoma (EES) is a rare tumor that affects a broad age distribution and can develop in various tissues throughout the body, with the presentation depending on the tissue affected [2]. Small bowel tumors are extremely uncommon to begin with, and sarcomas, often relegated to the ‘other’ category, comprise approximately 10 percent of them [3]. In general, small bowel tumors present with some constellation of symptoms including nausea, vomiting, bloating, constipation, diarrhea, or abdominal pain, while gastrointestinal bleeding is present in only 15 percent of cases [4]. Here, we discuss a case of EES of the small bowel that initially presented as gastrointestinal bleeding and was ultimately uncovered following a capsule endoscopy turned small bowel obstruction.

## Case presentation

**Figure 1:**
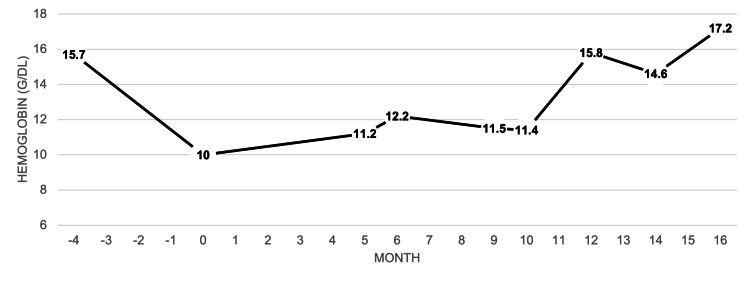
Hemoglobin trend Here, we graphically illustrate the changes in the patient's hemoglobin over the course of the case as it relates to his presentation to the clinic with melena at month 0.

**Figure 2:**
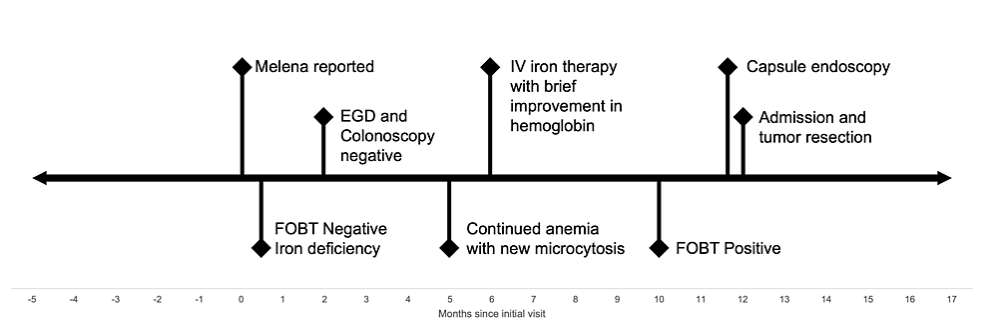
Timeline of events Outlined in this figure are the key events over the course of the patient's case. FOBT: fecal occult blood test; EGD: esophagogastroduodenoscopy; IV: intravenous

A 66-year-old man with a past medical history of perforated diverticulitis (status post hemicolectomy with colostomy formation and reversal 10 years prior) presented to the primary care office for evaluation of intermittent melena. The patient was otherwise asymptomatic and hemodynamically stable. Initial workup showed macrocytic anemia with hemoglobin 10.0, iron deficiency with ferritin of 7, negative fecal occult blood test (FOBT), and normal phenotype hemoglobin electrophoresis. Esophagogastroduodenoscopy (EGD) and colonoscopy were performed two months later with findings of Helicobacter pylori-negative gastritis and diverticulosis, respectively, but no source of active bleeding was identified. However, a follow-up complete blood count (CBC) three months after endoscopies showed continued anemia with new microcytosis, as displayed in Table 1. He was then referred to and seen by hematology/oncology, who initially gave the patient intravenous iron repletion with improvement in hemoglobin. However, when the anemia remained refractory, as demonstrated by Figure 1, they recommended capsule endoscopy for further investigation. Gastroenterology then saw the patient, obtained a positive FOBT, and agreed to proceed with capsule endoscopy, which found only jejunal and ileal erosions with no active bleeding and long small bowel transit time. Four days after swallowing the capsule, the patient began experiencing abdominal pain and reported that he still hadn’t noticed the capsule exit. Gastroenterology requested an abdominal radiograph for further investigation. However, the patient would ultimately present to the emergency room with worsening abdominal pain. Figure 2 highlights the pertinent events leading up to his hospital admission.

**Table 1:** Hemoglobin, MCV, and RDW throughout the case Displayed here is the patient's hemoglobin (Hgb), mean corpuscular volume (MCV), and red cell distribution width (RDW) from four months before presentation to the clinic with melena (month: -4), through his admission (month: 12), and in follow-up. Despite the improvement in hemoglobin at month 5, the patient's RDW becomes elevated and the MCV drops, consistent with iron deficiency.

Month	Hgb (g/dL)	MCV (fL)	RDW (%)
-4	15.7	98.7	12.2
0	10	100.3	11.8
5	11.2	76.3	18.7
9	11.5	88.9	17.2
10	11.4	88.1	16.8
12	15.8	91	14.9
13	14.6	94.1	14.7
15	17.2	93.3	13.9
Reference Range	13.2-17.1	80-100	11.0-15.0

In the emergency room, the patient reported worsening abdominal pain for the past four days. He also stated that he had not had a bowel movement or passed flatus for 24 hours. Physical examination was significant for moderate tenderness in the suprapubic abdomen. Abdominal radiograph identified a radiopaque foreign body in the left mid abdomen with a nonspecific small bowel gas pattern raising concern for obstruction. This was followed by computed tomography (CT) of the abdomen and pelvis showing a mildly dilated small bowel loop, concerning for early small bowel obstruction, with the presence of a metallic density and thickening of small bowel wall adjacent to the metallic density, consistent with capsule impaction. The general surgery service was consulted, and the initial plan was watchful waiting and supportive treatment with the hope that the patient would pass the capsule and relieve the obstruction. However, the patient’s abdominal pain continued to worsen, leading to the ultimate decision to proceed with exploratory laparotomy with plans to remove the foreign body and explore the gastrointestinal tract to identify the cause of persistent bleeding.

In the operating room, the patient was found to have a dilated, tense bowel with ischemic changes at a solitary site of severe small bowel obstruction site involving the proximal ileum, at which point the capsule was severely impacted. The involved bowel was densely adherent at the ostomy closure site involving the posterior rectus fascia, consistent with the bowel loop included within the suture closure of the fascia. The capsule was delivered via enterotomy, and the obstruction plus nonviable ileum were resected with the formation of a primary anastomosis. On postoperative day four, the patient required two units of packed red blood cell (PRBC) transfusion. Otherwise, the postoperative course was unremarkable, and the patient was discharged home on postoperative day six. Surgical pathology would later show involvement from serosa, muscularis, and submucosa into the mucosa by a round blue cell tumor. The resected tissue stained negative for CD34, CD117, desmin, S100, PLAP, synaptophysin, inhibin, and high molecular weight cytokeratin, while myogenic gave a background stain. On the other hand, the lesion stained positive for vimentin, BCL2, CD99, and cytokeratin (weakly). At this point, further stains were necessary to differentiate between synovial sarcoma and EES. TLE1 would return negative and FLI-1 would return positive, which was more consistent with EES. Thus, the patient was referred to a comprehensive cancer center for further management. As the patient appeared to have complete resection of localized disease, the decision was made to follow up with surveillance CT scans, initially every three months. At the time of this report, the patient remains without evidence of disease four years later.

## Discussion

Current guidelines from the American Academy of Family Physicians (AAFP) for suspected upper gastrointestinal bleeding in a hemodynamically stable patient suggest performing EGD. If no source is found on EGD, further work-up should be guided based on clinical judgment and patient presentation [5]. Obscure gastrointestinal bleeding is defined as recurrent bleeding from an unidentified source after upper endoscopy and colonoscopy and can be a difficult problem to address [6]. Obscure gastrointestinal bleeding is most often due to inflammatory processes of the distal small bowel secondary to pathologies such as malignancy, angiodysplasia, diverticula, and celiac disease [6]. In our patient’s case, bleeding was likely intermittent from the EES of the proximal ileum, explaining the intermittently positive FOBT. For diagnosis, such pathologies require extended enteroscopy or capsule endoscopy, as these two methods allow for direct visualization of the distal small bowel. Capsule endoscopy has proven extremely valuable for cases of suspected small bowel pathology, as evidenced by the relatively high diagnostic yield of small bowel tumors in these patients [7]. While obstruction certainly was not the intent of the capsule endoscopy in our patient, the failure of the capsule to pass ultimately led to this patient’s diagnosis.

Of all neoplasms involving the gastrointestinal tract, small bowel tumors comprise only 1-2 percent [8]. While Ewing sarcomas typically present in the bones of older children and young adults, they can also develop in extraskeletal locations in 20-30 percent of cases [9]. The clinical manifestations of EES depend on the site of the tumor and/or metastases, ranging from asymptomatic to localized pain, or gastrointestinal bleeding as was seen in this patient’s case. Due to its rarity, EES currently lacks explicit treatment guidelines. Single case and cohort studies have suggested a similar treatment approach as to traditional Ewing sarcomas - definitive surgery for localized disease and chemoradiation followed by surgical resection for metastatic disease [9]. As such, our patient’s disease was managed through surgical resection alone followed by surveillance, and he has been without evidence of disease for four years. Striefler et al. found in their cohort study that comorbidities were more strongly predictive of outcomes rather than older age in adult patients [10]. In children, EES has interestingly been shown to carry a more favorable prognosis than its classic skeletal counterpart [11]. As more cases of EES are discovered and reported, larger analyses can be completed to assess treatment outcomes and provide recommendations.

## Conclusions

This patient’s case highlights the importance of persistent investigation of refractory iron deficiency anemia. In cases with concern for gastrointestinal bleeding in the setting of negative upper and lower endoscopies, direct examination of the distal small bowel is paramount. Capsule endoscopy is relatively convenient for patients and providers and can provide the necessary answers in cases of obscure gastrointestinal bleeding. Although there is a minimal risk of bowel obstruction, this risk is far outweighed by the benefit of the potential detection of a nefarious diagnosis.
